# Supplementary figures and images for: Maternal total sleep deprivation causes oxidative stress and mitochondrial dysfunction in oocytes associated with fertility decline in mice
Source: PLoS One. 2024 Oct 16;19(10):e0306152. doi: 10.1371/journal.pone.0306152 (PMC11482706; doi:10.1371/journal.pone.0306152)

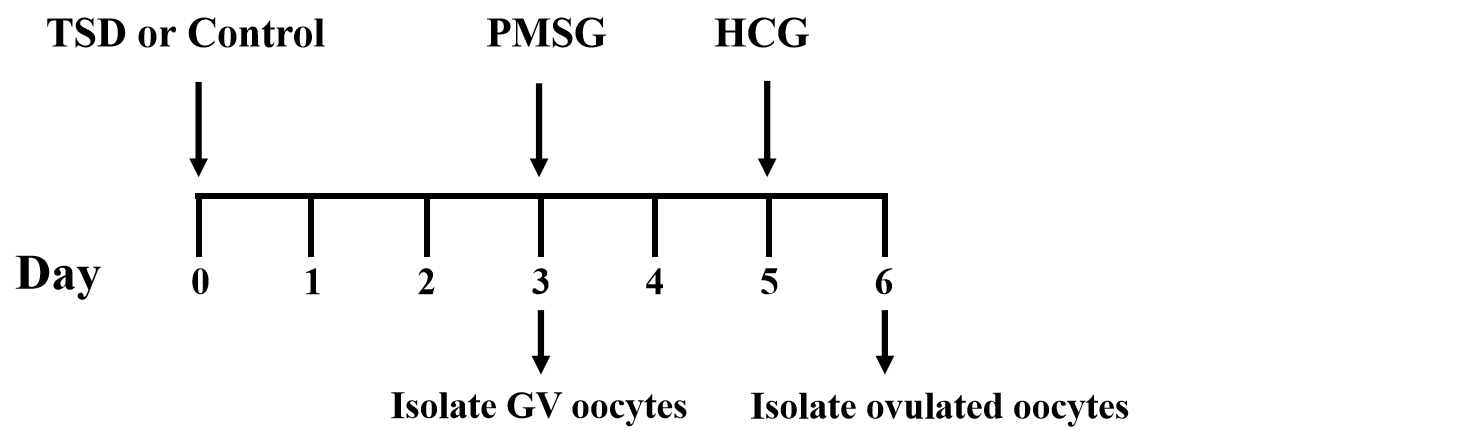

Supplement: S1 Fig — (A) Female mice were deprived of sleep via forced locomotion on day 0. There days later, part of the mice were identified by the stage of estrous and taken for blood immediately. Part of the mice were sacrificed to collect the GV oocytes. To retrieve ovulated oocytes for following molecular, cellular and biochemical analysis, part of the mice were administrated with 10 IU PMSG (d 3). These mice were injected with 10 IU HCG 2 d after PMSG. At 13.5 h after HCG administration, oocytes were collected from oviductal ampullae (d 6). (TIF) [file pone.0306152.s001.tif]

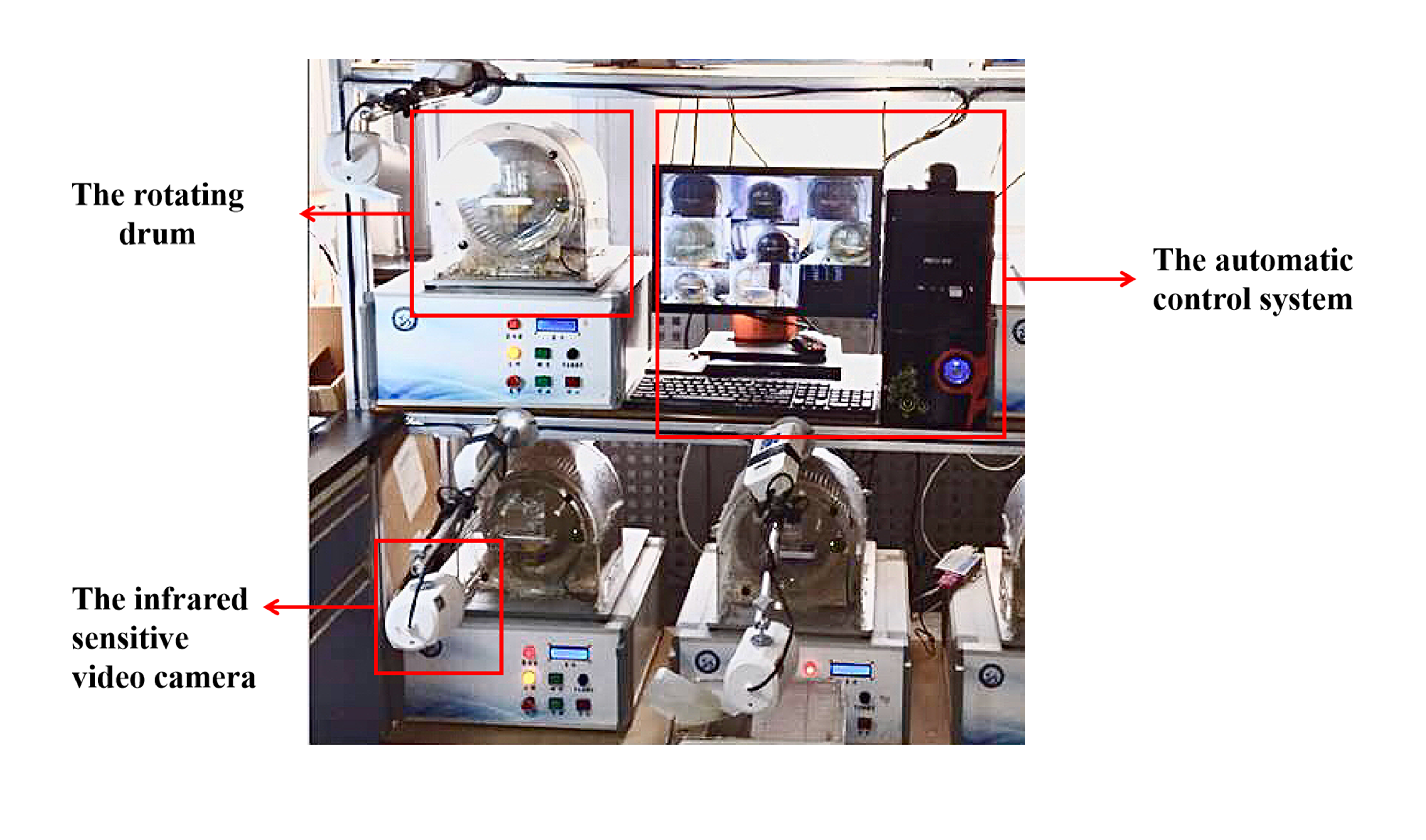

Supplement: S2 Fig — The drums were large, motorized, equipped with stainless-steel activity wheels with 22 cm outside diameter and 18 cm internal wheel width. The wheels were run through a computer-controlled motor mediated by a drive strip. The front and back panels of the drum are made of Plexiglas. A water bottle and a feeding RACH were mounted on the front panel. The lens of an infrared sensitive video camera was mounted 20 cm in front of the wheel. There was video surveillance throughout the deprivation. (TIF) [file pone.0306152.s002.tif]
