# Supplementary material for: Maternal total sleep deprivation causes oxidative stress and mitochondrial dysfunction in oocytes associated with fertility decline in mice
Source: PLoS One. 2024 Oct 16;19(10):e0306152. doi: 10.1371/journal.pone.0306152 (PMC11482706; doi:10.1371/journal.pone.0306152)
Supplement: S1 Table — Gapdh was chosen as the reference gene. The primers used for the amplification of Gapdh fragment are listed as follows, Forward: 5’-CCCCAATTGTGTCCGTCGTG-3’; Reverse: 5’-TGCCTGCTTCACCACCTTCT-3’. (DOCX) [file pone.0306152.s003.docx]

**Table S1. Sequences of primers for the internal reference gene and genes. F: Forward; R: Reverse.**

| **Gene name** | **Abbreviation** | **Primers 5’-3’** |
| --- | --- | --- |
| **Aryl Hydrocarbon Receptor Nuclear Translocator Like 2** | **Arntl2** | **F:GGTTGGATGCGAAAGAGGGA**  **R:ATCGTCTGGAGCCAGAATGC** |
| **AT-rich interaction domain 5B** | **Arid5b** | **F:CAGAACGTCGAGATGGAGCC**  **R:ATTTGGCGCGAACTCATCAC** |
| **ATP synthase, H+ transporting,**  **mitochondrial F1F0 complex, subunit E** | **Atp5k** | **F:GTTCAGGTCTCTCCACTCATCA**  **R:CGGGGTTTTAGGTAACTGTAGC** |
| **Cell dividing cyclin 2** | **Ccnd2** | **F:ACCTCCCGCAGTGTTCCTATT**  **R:CACAGACCTCTAGCATCCAGG** |
| **CD200 molecule** | **Cd200** | **F:TGAGCACAGCTCAAGTGGAA**  **R:TGTCTTTGTAGGCAGGCTGG** |
| **Meiotic nuclear divisions 1** | **Mnd1** | **F:GGGGAGGAAAAGAGAACCCG**  **R:ACCGTCATCCACTAGGCTCT** |
| **Proline rich 18** | **Prr18** | **F:TTTCTAACGCCTCACCACCC**  **R:CATTTCACAGGTCCTCCCCC** |
| **Pregnancy specific glycoprotein 17** | **Psg17** | **F:AGTGAGTGAGCAGTGCTGTG**  **R:TAAGAGGGAGGCTGTGAGCA** |
| **Ribosomal protein L38** | **Rp138** | **F:CCCCGTTCTCTTCGGTTCTC**  **R:ACAGACTTGGCATCCTTCCG** |
| **Tropomyosin 3** | **Tpm3** | **F:TGGACCAGAACCTGAAGTGTC**  **R:CAGGGATGGGGAGGTCTACA** |
| **Up-regulated during skeletal muscle growth protein 5** | **Usmg5** | **F:GTTTGGGGTTCGGACGAAGA**  **R:CAGCAAAGCAATGCCTCCAT** |
| **Zinc finger protein 984** | **Zfp984** | **F:GCTGGCCTCCTAGGACATTGA**  **R:CAGGGTCTCTGTGGAGTGTTTA** |
