# Supplementary material for: Maternal total sleep deprivation causes oxidative stress and mitochondrial dysfunction in oocytes associated with fertility decline in mice
Source: PLoS One. 2024 Oct 16;19(10):e0306152. doi: 10.1371/journal.pone.0306152 (PMC11482706; doi:10.1371/journal.pone.0306152)
Supplement: S1 File — (DOCX) [file pone.0306152.s004.docx]

**Data set**

**Figure1.** The effects of sleep deprivation on maternal weight and sex hormone levels.

(**A** and **B**) Body weights at the beginning and body weight changes from beginning to termination of the sleep deprivation experiment in the stationary group, the TSD group and the forced movement group.

| Sedentary | TSD | Forced activity |
| --- | --- | --- |
| 30.00 | 30.00 | 29.8 |
| 28.30 | 27.90 | 25.8 |
| 27.00 | 32.20 | 26.8 |
| 27.60 | 26.80 | 28.8 |
| 29.40 | 27.80 | 26.2 |
| 26.80 | 28.80 | 27.6 |
|  | 27.80 | 33.1 |

| Sedentary | TSD | Forced activity |
| --- | --- | --- |
| 0.10 | 0.10 | 6.4 |
| 0.10 | 0.10 | 6.2 |
| -2.50 | 7.40 | -2.4 |
| -0.90 | 0.60 | 3.1 |
| 7.00 | 1.20 | 6.0 |
| -0.80 | 3.20 | -2.4 |
| 0.00 | 6.60 | 3.1 |
| 3.10 | -1.90 |  |
| 6.30 | 4.00 |  |
| 9.00 | 5.10 |  |
| 4.50 |  |  |
| 3.70 |  |  |
| 4.80 |  |  |

**(C** and **D**) Concentrations of serum estrogen (C) and FSH (D) during four stages of estrus cycle in the stationary, forced night activity, and TSD groups.

| estrogen | TSD | | | Stationary | | | forced night activity | | |
| --- | --- | --- | --- | --- | --- | --- | --- | --- | --- |
| Diestrus | 37.000 | 2.519 | 3 | 50.416 | 2.307 | 3 | 52.75 | 1.690 | 3 |
| Proestrus | 59.235 | 2.210 | 3 | 82.635 | 6.629 | 3 | 91.28 | 6.629 | 3 |
| Estrus | 47.130 | 2.210 | 3 | 65.715 | 1.690 | 3 | 61.28 | 2.307 | 3 |
| Metaestrus | 15.420 | 2.197 | 3 | 44.122 | 3.389 | 3 | 32.75 | 3.389 | 3 |

| FSH | TSD | | | Stationary | | | forced night activity | | |
| --- | --- | --- | --- | --- | --- | --- | --- | --- | --- |
| Diestrus | 2.605 | 0.251900 | 3 | 2.493333 | 0.230700 | 3 | 2.300000 | 0.130700 | 3 |
| Proestrus | 3.010 | 0.221000 | 3 | 4.105000 | 0.338000 | 3 | 4.605000 | 0.338000 | 3 |
| Estrus | 2.810 | 0.221000 | 3 | 3.326667 | 0.669000 | 3 | 3.339000 | 0.369000 | 3 |
| Metaestrus | 1.815 | 0.219700 | 3 | 2.300000 | 0.162900 | 3 | 2.200000 | 0.138000 | 3 |

**Figure 2.** The total sleep deprivation (TSD) mice ovulated normal number of eggs but showed impared early embryo development .

1. The average number of ovulated eggs. Four female mice with plugs were examined for each group.

| Sedentary | TSD | Forced Activity |
| --- | --- | --- |
| 16.00 | 21.00 | 15. |
| 15.00 | 20.00 | 18. |
| 12.00 | 29.00 | 17. |
| 18.00 | 7.00 | 11. |
| 16.00 | 14.00 | 13. |
| 13.00 | 12.00 | 15. |
| 17.00 | 12.00 | 19. |
| 18.00 | 16.00 | 12. |
| 10.00 | 16.00 | 15. |
| 13.00 | 11.00 | 18. |
| 16.00 | 14.00 |  |
|  | 15.00 |  |
|  | 9.00 |  |
|  | 7.00 |  |
|  | 14.00 |  |
|  | 11.00 |  |
|  |  |  |

1. The fertilization rate for the stationary group, the TSD group and the forced movement group. The data are expressed as means± SEM of at least three replicates. Different letters indicate statistically significant difference (P<0.05).

(**C**) The blastocyst rates of fertilized eggs after in vitro culture (IVC) for 5d.

| Sedentary(%) | Forced Activity(%) | TSD(%) |
| --- | --- | --- |
| 83.7500 | 72.7300 | 50.0000 |
| 53.3300 | 100.0000 | 46.0000 |
| 91.6700 | 100.0000 | 31.0000 |
| 88.8900 | 100.0000 | 41.0000 |
| 80.0000 | 50.0000 | 100.0000 |
| 80.0000 |  | 29.0000 |
|  |  | 50.0000 |
|  |  | 85.0000 |

**Figure 3.** Single-GV oocyte RNA sequencing.

**Figure 4.** Effects of total sleep deprivation on ROS in the GV and MII oocytes.

(B)The fluorescence intensity of the carboxy-H2DCF fluorescence of each GV oocyte in the stationary, the TSD and the forced night activity groups were quantified using ZEN (2012) software. Data are expressed as mean ± SEM of at least 3 independent experiments and 3 mice were killed to obtain a minimum of 50 oocytes for each experiment. Different letters indicated statistically significant differences (P<0.05).

|  | control | sleep deprivation | forced activity control |
| --- | --- | --- | --- |
| mean | 1519.09 | 2448.74 | 1661.73 |
| SEM | 321.494 | 612.110 | 536.682 |

(C)Effects of total sleep deprivation on ROS production measured by the carboxy-H2DCF fluorescence of each of the MII oocytes using ZEN (2012) software. Data are mean ± SEM of at least 3 independent experiments. Four superovulated mice were killed to obtain a minimum of 30 oocytes for each experiment. Different letters indicate statistically significant differences (P<0.05).

|  | control | sleep deprivation | forced activity control |
| --- | --- | --- | --- |
| control | 1505.85 | 2547.27 | 1321.44 |
| SEM | 405.713 | 459.174 | 196.006 |

**Figure 5**. Effects of sleep deprivation on the mtDNA copy number in single GV and MII oocytes. Single oocyte was extracted and used for determining the mtDNA copy number.

As shown in Figure 5A, GV oocytes in the TSD group contained more mtDNA copies compared to those in the stationary and the forced night activity groups **(474600±23000 versus 317300±29240 and 362800±32150 , control; P<0.05)** . However, the average mtDNA copy number in MII oocytes from the TSD mice was significantly decreased compared with that of the MII oocytes from the stationary control and the forced night activity control groups **(303300±22680 versus 390000±25830 and 427300±43440 , control; P<0.05)** .

**Figure 6**. Maternal sleep deprivation disrupts mitochondrial redistribution in the GV and MII oocytes. The oocytes were stained with MitoTracker-Red to detect the mitochondrial distribution patterns, and chromosomes were counterstained with Hoechst 33342 (blue) to confirm meiotic stages.

1. In the GV oocytes, three different distributions of mitochondria were observed: (i) perinuclear distribution, (ii) homogeneous distribution and (iii) clustered distribution. Bar=20μm.

**
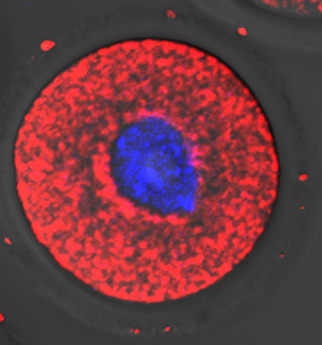

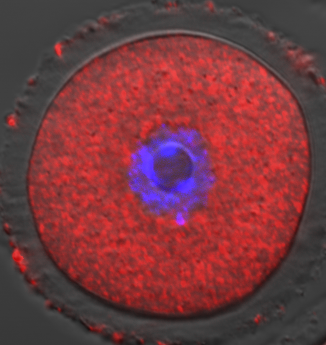

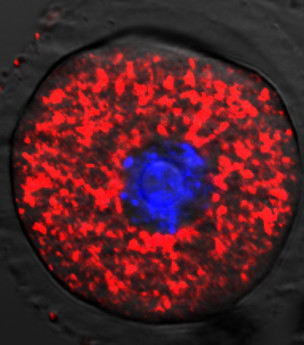
**

**
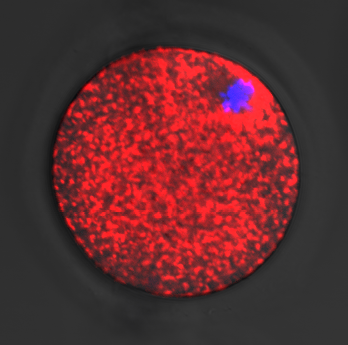

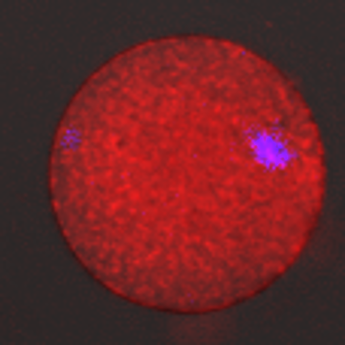

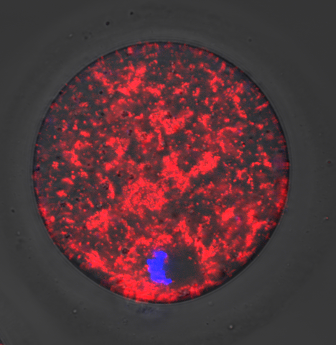
**

| Sedentary  (i) | Sedentary  (ii) | Sedentary  (iii) | TSD  (i) | TSD  (ii) | TSD  (iii) | Forced activity(i) | Forced activity(ii) | Forced activity(iii) |
| --- | --- | --- | --- | --- | --- | --- | --- | --- |
| 70 | 25 | 5 | 42 | 30 | 27.5 | 65 | 29 | 6 |
| 1.15 | 2.31 | 1.15 | 2.31 | 5.77 | 6.35 | 5.77 | 3.46 | 2.31 |

**(B)** Quantification of the GV oocytes with each mitochondrial distribution pattern from the stationary, TSD and forced movement groups.

(D)Proportions of MII oocytes from stationary, TSD and forced night activity groups to show each mitochondria distribution pattern described in C. Data in B and D are expressed as mean ± SEM of at least

3 independent experiments.

| Sedentary  (i) | Sedentary  (ii) | Sedentary  (iii) | TSD  (i) | TSD  (ii) | TSD  (iii) | Forced activity(i) | Forced activity(ii) | Forced activity(iii) |
| --- | --- | --- | --- | --- | --- | --- | --- | --- |
| 64.5 | 28.09 | 7.865 | 31.65 | 44.335 | 24 | 65 | 29 | 6 |
| 6.35 | 2.21 | 2.47 | 1.91 | 2.70 | 4.62 | 8.08 | 3.3 | 2.32 |

**Figure 7**. Effects of sleep deprivation on the mitochondrial transmembrane potential in the GV and MII oocytes.

**
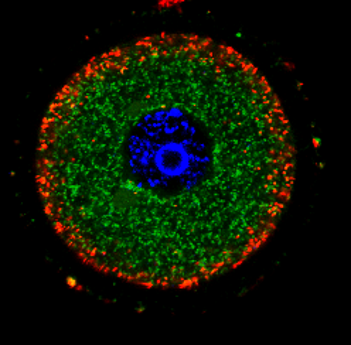

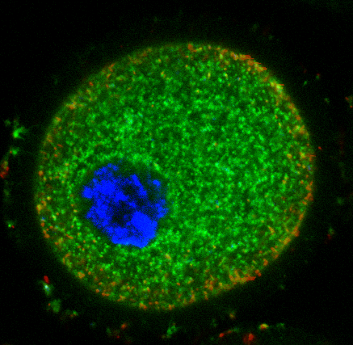

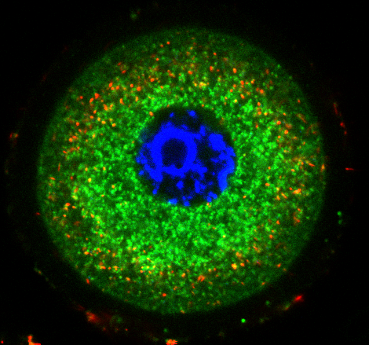
**


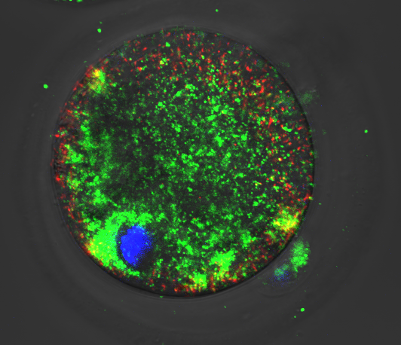

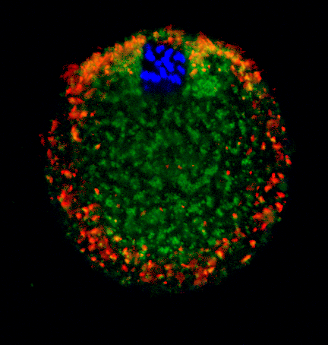

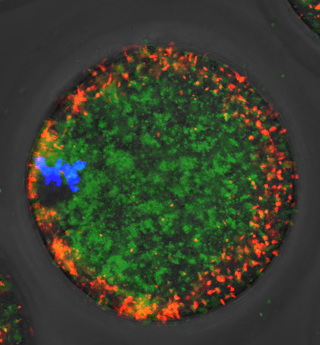


1. The ratio of red/green fluorescence intensities in the GV oocytes from the stationary, TSD and forced night activity groups were quantified as an indicator of mitochondrial activity. The data represents means ± SEM of at least three independent experiments. Three mice were killed to obtain a minimum of 50 GV oocytes for each replicate experiment. Different letters denote statistically significant differences (P<0.05).

|  | Sedentary | TSD | Forced activity |
| --- | --- | --- | --- |
| mean | 0.567841086 | 0.752855157 | 0.566187565 |
| sem | 0.02046649649 | 0.04129504480 | 0.02028928287 |

(D) The ratio of red/green fluorescence intensity in ovulated MII oocytes was examined in all groups. The data represents means ± SEM of at least three independent experiments.

| Sedentary | TSD | Forced activity | Sedentary |
| --- | --- | --- | --- |
| mean | 1.032895400 | 1.032895400 | 1.052901 |
| sem | 0.2018284694 | 0.2018284694 | 0.139413 |

**Figure 8.** Maternal sleep deprivation leads to defective spindles and misaligned chromosomes.

**(A)** Representative images of the spindle and chromosomes in stationary, TSD and forced night activity groups. MII oocytes from the stationary and the forced movement mice present a normal spindle and aligned chromosomes. In the TSD mice, the MII oocytes exhibited various morphologically abnormal spindles and misaligned chromosomes.


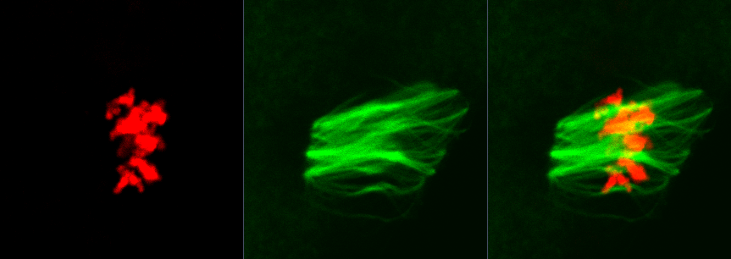


**
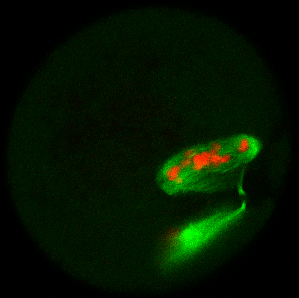

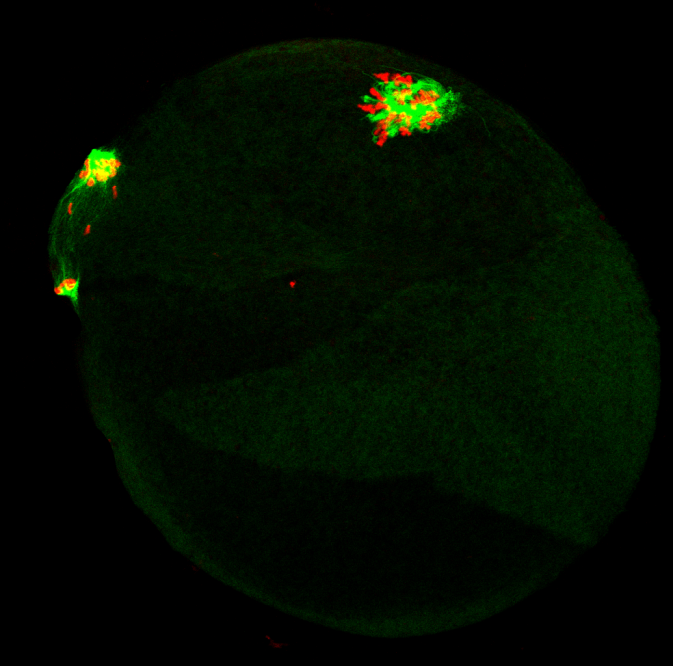

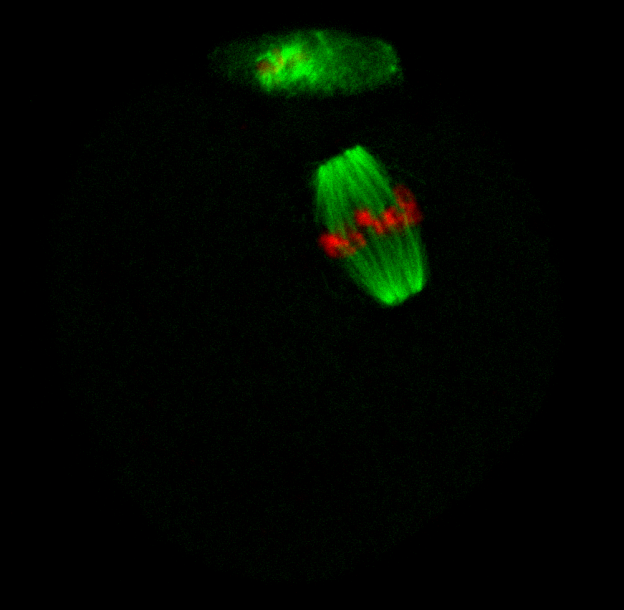

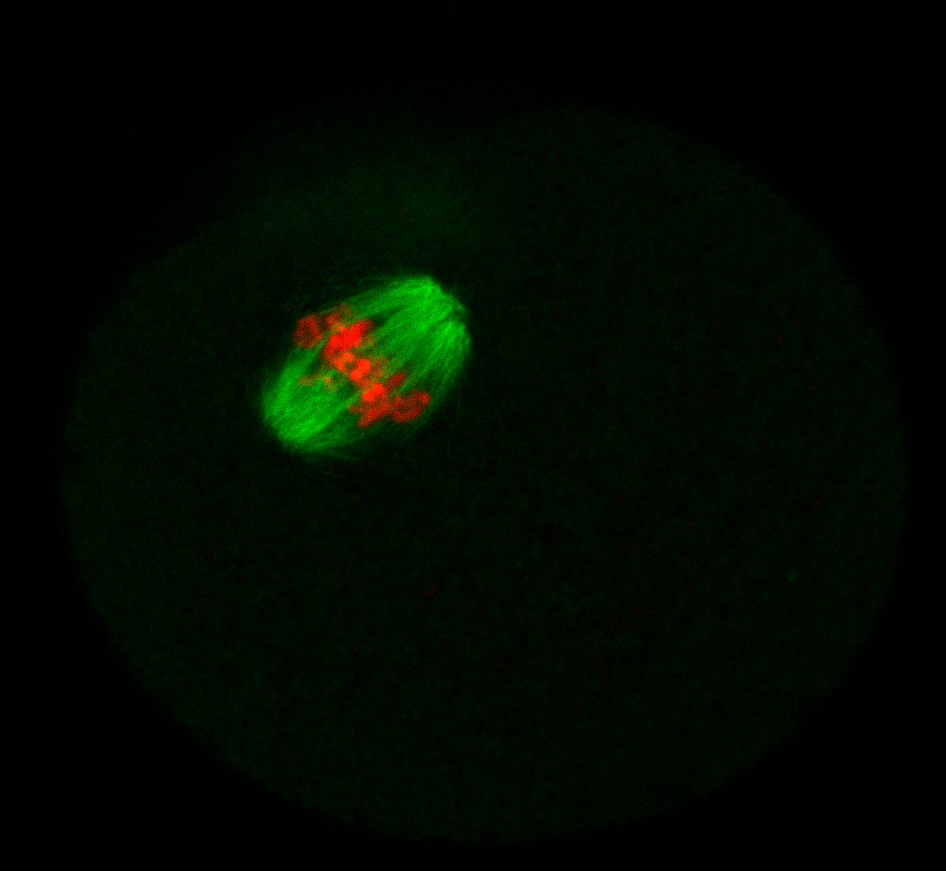
**

**(B)** Percentages of oocytes with abnormal spindles and misaligned chromosomes in MII oocytes from the stationary, TSD and forced night activity groups. Data are presented as means±SEM of 3 independent experiments. Different letters denote statistically significant differences (P<0.05).

|  | **Spindle defects** | | **Chromosome misalignment** | |
| --- | --- | --- | --- | --- |
|  | **mean** | **sem** | **mean** | **sem** |
| Sedentary | 3.875 | 0.97 | 2.2 | 0.67 |
| TSD | 8.025 | 1.52 | 6.3 | 1.35 |
| Forced activity | 4.375 | 0.98 | 2.95 | 0.66 |
